# Experimental Assessment of Thermal Performance and Bridging Effects of Low-Cost Sandwich Panels under a High-Temperature Impinging Jet

**DOI:** 10.3390/ma13163620

**Published:** 2020-08-16

**Authors:** Wei Ye, Jian Cai, Yixiang Huang, Chengqiang Zhi, Xu Zhang

**Affiliations:** 1 School of Mechanical Engineering, Tongji University, Shanghai 201804, China; cj@tongji.edu.cn (J.C.); 1830255@tongji.edu.cn (Y.H.); 1810798@tongji.edu.cn (C.Z.); xuzhang@tongji.edu.cn (X.Z.); 2 Key Laboratory of Performance Evolution and Control for Engineering Structures of Ministry of Education, Tongji University, Shanghai 200092, China

**Keywords:** shipbuilding, insulation material, fabrication, fastener, heat flux, thermal bridge

## Abstract

Sandwich panels are commonly used across industries for their ability to bear structural and thermal loads. In this paper, a panel chamber matching apparatus was designed to investigate the thermal performance of eight steel-based panels by exposing them to an impinging jet at approximately 550 °C for 30 min. Three types of low-cost materials (polycrystalline filaments, silica aerogel, and aluminum silicate) were used as the insulation core. The temperature of the panel surfaces was measured, as well as the metallic fasteners, including bolts, nails, battens, seams, and angle iron, to examine their thermal bridge effects. Major conclusions include the following: first, the maximum temperature on the impinged surface was consistent among all 20 cases, whereas that of the surface under free convection varied, ranging from 41 to 120 °C, depending on the core and thermal bridges. Second, most of the highest temperatures on opposite surfaces were caused by a section of bare angle iron, and this bridging effect could be significantly reduced by up to 50 °C using a few layers of cloth, although the improvement could be temporary. Bolts and nails were less effective as thermal bridges, while the battens could be more effective. Third, the estimated heat flux of all specimens ranged from 167 to 331 W·m^−2^.

## 1. Introduction

A sandwich panel, by definition, consists of a low-density core and one thin stiff layer bonded to each side [1,2]. Considering the benefits of lightweight rigid structures that can also serve as thermal insulation, sandwich panels have been popular in aeronautic and astronautic engineering for decades [3,4]. While aluminum alloy, steel, laminated composites, and other materials can be used for the outer layers to bear the structural load [5,6], stochastic cellular materials (SCMs) and periodic cellular materials (PCMs) are widely applied for the core to bear the thermal load [7,8]. A sandwich panel with a metallic honeycomb core, a common example of PCM, is one of the most weight-efficient structures and can be extensively found in commercial aircraft [9,10,11,12]. In recent years, another group of PCMs, lattice structures, e.g., tetrahedral, pyramidal, Kagome, woven textile, and X-type lattices, have increasingly drawn attention, mostly owing to the open topology with embedded additional benefits [3,13,14,15,16,17]. For instance, the cavities are possible coolant channels, while the lattice core can sustain mechanical loads [18,19]. For hypersonic aircraft, advanced sandwich panels using composite materials can also be incorporated into the thermal protection system to protect the vehicle from overheating [20,21].

Overall, apart from aerospace applications, sandwich panels are also in demand in building construction (where they are generally referred to as structural insulated panels or SIPs), refrigeration (e.g., cold stores and refrigerated warehouse facilities), automobiles, and marine uses, among others [22,23,24]. There is no one-size-fits-all solution. A larger variety of materials, from natural to man-made, metallic to non-metallic, and single to composite, are being assembled to achieve high strength-to-weight ratios in sandwich panels, while the products exhibit diverse functions. Nonetheless, two interlinked issues are key to the application of sandwich panels: thermal and physical performance, and manufacturing [3]. A brief discussion of the two issues is given below.

For construction, SIPs can be prefabricated and used in walls, floors, and roofs, as part of the building envelope. On top of structural support and thermal insulation for a building, which is designed to stand for a long time, SIPs also act as air, vapor, and acoustic barriers [25,26,27]. In a building, the indoor temperature is normally kept in (or slightly outside of) a thermal comfort zone [28]. The temperature difference between the two sides of SIPs usually falls within 15 °C in summer, depending on the local climate. For an extremely cold region, indoor–outdoor temperature differences can reach more than 60 °C in winter [26]. However, the differences are still small compared to those in aerospace applications, for example. As a result, long-lasting, low-cost, eco-friendly, and thermal-resistant materials are preferred (e.g., hemp–lime composite materials have shown promising thermal performance to be an alternative for other commonly used construction materials [29]). At present, structural boards are commonly made of sheet metal, cement, or wood-based materials such as plywood or oriented strand board (OSB), to name a few, and the insulating core is often made of synthetic foam, e.g., expanded polystyrene (EPS), extruded polystyrene (XPS), polyisocyanurate (PIR), polyurethane (PU), or other phase-change materials [25,26,27,30]. Similarly, in the refrigeration industry, high-density polymeric foam core (e.g., PU) with two sheets of galvanized steel is a reasonable choice for insulating cold rooms.

By and large, when light weight is needed and the outside temperature or service temperature is much higher, the sandwich panel core requires proper geometric considerations. Corrugation, honeycomb, and lattice have all been extensively studied [20,31,32,33,34]. By using additional inner insulation materials or coolants, high service temperatures can be achieved. The service temperature of carbon fiber reinforced polymers with corrugated NOMEX honeycomb and lattice core sandwiches is usually below 400 °C [35,36,37]. A titanium alloy corrugated sandwich is capable of sustaining 700 °C [38]. A metallic honeycomb core sandwich has been proven to have adequate thermal performance at 900 °C [31]. ZrO_2_ and ZrB_2_ ceramics and carbon fiber-reinforced silicon carbide (C/SiC) with corrugate or lattice core sandwiches have high service temperatures up to 1000–1600 °C [20,39,40]. Although the thermal performance when employing these technologies is enhanced, they can be constrained by the manufacturing process for at least the following reasons. First, the production cost to make lattice or honeycomb can be higher than that of polymeric foams, not to mention the additional materials required to integrate into the core [15,41]. Various fabrication methods are available, and some waste materials [3]. Second, when the integrated materials are not rigid under service temperature, the lattice acts as the supporting frame. For example, aerogel-filled sandwich panels can be employed as building insulation [42]. Caution is needed when selecting the lattice material, considering the stiffness and cost, or it could behave like a thermal bridge.

In practice, there is a need to develop affordable sandwich panels that can sustain relatively high temperatures. One application is in a confined parking garage housing heavy vehicle that release high-temperature diesel exhaust, which also sometimes has a high airflow rate [43]. Another example is in the labor-intensive shipbuilding industry, where sandwich panels can be standardized into modules and put into deck and wall sections at a large scale. Besides saving labor, the almost deformation-free sandwich elements are post-treatment-free [44]. However, novel designs of low-cost sandwich panels with high thermal resistance are still in demand, especially for sections with a heating source at a short distance, e.g., an exhaust vent of a combustion gas turbine. Thermal bridges can be an issue because metallic materials are still heavily used in structural panels for ships.

In this study, we set up a panel chamber matching experimental apparatus to investigate the thermal performance of eight steel-based sandwich panels while being exposed to a high temperature of approximately 550 °C in the form of an impinging jet from a natural gas burner at its upper limit. The sandwich panels were designed to be used in cargo ships in large quantities to endure fire, as well as intentional or unintentional short-distance heating sources. As a matter of common practice, steel was used on one side to blend in with the monolithic structure, while the material on the other side, which would be under direct heating, was mainly stainless steel. Ordinary insulation materials that were all flexible and low cost were used as the core. Metallic fasteners were used as the binding strategy and the effects of thermal bridges were evaluated. All the testing modules were manufactured in a workshop using raw materials and fabricating techniques similar to those used in real practice.

## 2. Materials and Methods

### 2.1. Materials and Fabrication of Sandwich Panels

**Figure 1:**
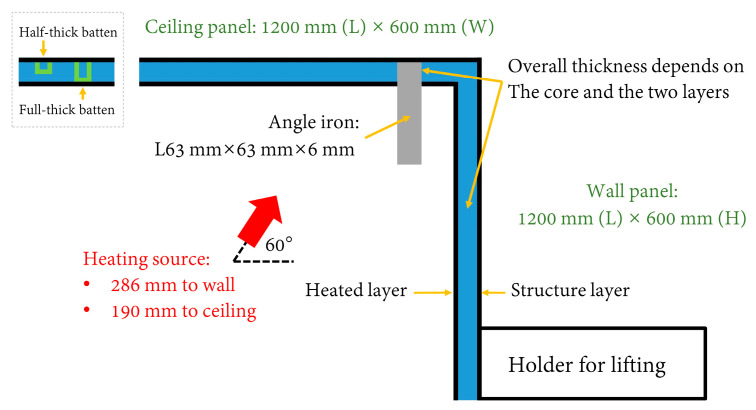
Common shapes and dimensions of specimens (side view).

**Figure 2:**
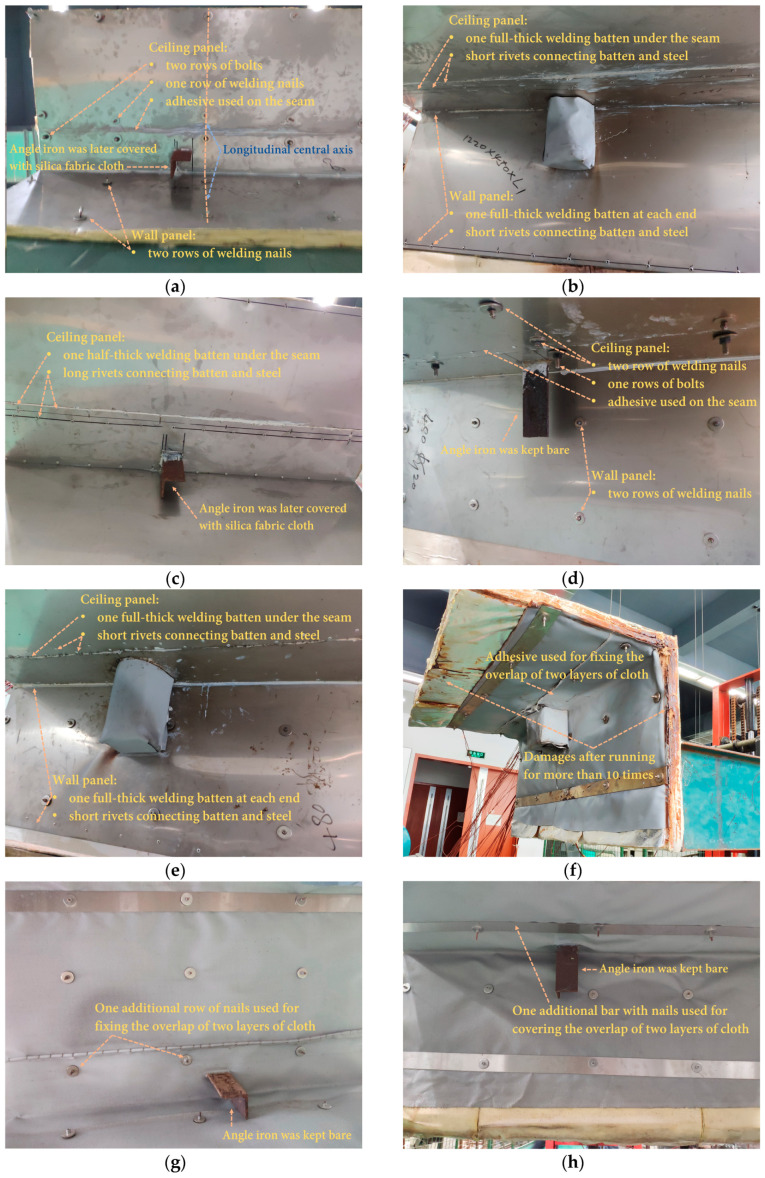
Images of sandwich specimens at different stages of the experiment: (**a**) Specimen 1 prior to testing; (**b**) Specimen 2 after two tests; (**c**) Specimen 3 prior to testing; (**d**) Specimen 4 after two tests; (**e**) Specimen 5 prior to testing; (**f**) Specimen 6 after testing more than 10 times, mostly pre-experiment, and lifted by a forklift; (**g**) Specimen 7 after two tests; and (**h**) Specimen 8 after two tests.

The sandwich materials subjected to testing were designed by following two principles, considering the potential to be produced in a modular manner. First, the insulation materials making up the core should be, by balancing thickness, low-cost and lightweight. Second, the fabricating techniques and methods (including fastening) should not be time-consuming. As a result, 7 mm thick steel of grade EH36 was used as the stiff layer on one side to represent the ship structure. For five out of the eight specimens, 1 mm stainless steel was on the other side, while in the other three cases, the material for the heating layer was further reduced to a layer of silica fabric cloth with a few stainless-steel bars for compacting. Three types of commonly found insulation products for ships were selected as the core: (1) 20 mm thick polycrystalline filaments, which are produced with various crystal ores as raw materials, e.g., alumina (Al_2_O_3_), silica (SiO_2_), zirconia (ZrO_2_), etc.; (2) 10 mm thick silica aerogel; and (3) 20 mm thick aluminum silicate (*x*Al_2_O_3_·*y*SiO_2_·*z*H_2_O). Depending on heat resistance, weight, and cost, two layers of insulation were used for all cores. As of this writing, aerogel was still the most expensive among the three insulation materials; therefore, instead of proposing a 40 mm thick aerogel core, a 20 mm thick core was used to keep the panel affordable. In addition, various fasteners were used to hold up the specimens, including in-core fasteners, e.g., battens (with rivets); penetrating connectors, e.g., bolts and welding nails; and compacting bars. As shown in Figure 1, the main body of the specimens was L-shaped so as to model a corner, consisting of a ceiling panel and a wall panel. For practical reasons, the wall panel was extended by a specially designed holder, which can be used by a forklift. A 20 cm long angle iron, which was welded with the structure layer and also investigated for its thermal bridge effect, was attached to the ceiling to represent a hanging bracket for pipes in a real ship. Components or accessories on the panels were normal in practice. Additional information on the materials and fabrication methods used to produce the specimens can be found in Table 1. Images of the specimens at different stages of the experiment are shown in Figure 2. Many of the potential thermal bridges, e.g., bolts, nails, seams, and a section of angle iron, were visible, and annotations are given in Figure 2. Battens were hidden in the panel core, and illustrations are included in Figure 1 as well.

### 2.2. Experimental Setup and Heating Source

**Figure 3:**
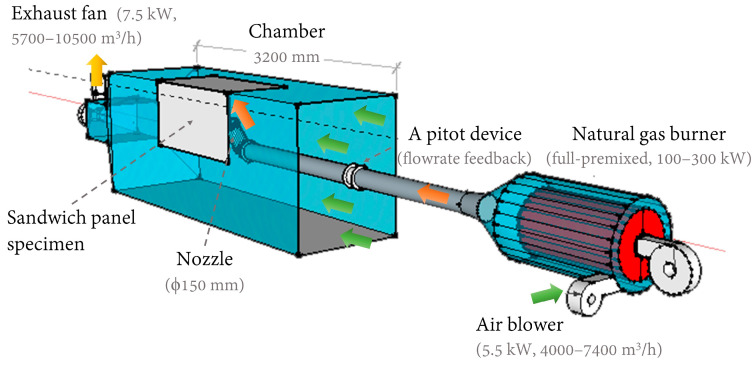
Schematic diagram of the experimental setup.

The thermal performance of the proposed sandwich panels was tested by an experimental apparatus, which mainly consisted of a chamber, a full-premixed natural gas burner with a high-pressure air blower, and an exhaust fan connected to outdoor air, as shown in Figure 3. Both the exhaust fan and air blower were controlled by a variable-voltage/variable-frequency (VVVF) drive system.

The major body of the chamber was 3.2 m long, with a cross-section of 1.2 m (H) × 1.0 m (W). As shown in Figure 3, an opening was cut from the chamber to match the specimen. The dimensions of the opening were slightly larger than those of the specimen, or it would be difficult to fit the two rigid pieces together. However, narrow gaps between the edges of the panel and the chamber were still available when some of the specimens were in position. At the edges, the core was not enclosed with steel to protect it from direct heating (see Figure 2f for evidence), because the chamber was kept at negative pressure overall, due to the exhaust fan. Precautions were taken to ensure the safety of the personnel, e.g., once the specimen was fitted, the forklift was standing by in situ at all time to make sure it would not fall from the chamber.

The natural gas burner can produce high airflow, i.e., an impinging jet toward the panel, at high temperature using municipally supplied gas with assistance from the air blower and a controlling system (including a pitot device to provide airflow rate feedback). In this study, we adjusted the impinging temperature to the upper limit at approximately 550 °C to fully test the thermal performance and possible drawbacks, e.g., severe thermal bridge, efficiency loss in some part of the panel, etc. As part of the controlling system, the gas temperature was measured in the gas pipe before exiting the nozzle. The corresponding air velocity at the exit of the nozzle (V) was not directly measured, but instead was determined at around 80–90 m/s based on the gas temperature and estimated fume density.

As indicated in Figure 1, to cover potential thermal bridges (e.g., angle iron, bolts, welding nails, battens, etc.) of the two panels simultaneously, the inclination angle of the nozzle was set at 60° (to focus more on the angle iron, and a requirement of the industry), and the center of the nozzle outlet was approximately 286 mm and 190 mm away from the wall and ceiling panels, respectively.

Two longitudinal positions of the nozzle were adjusted throughout the experiment. The first position was aimed at the longitudinal center of the specimen. The angle iron in most cases was approximately 70–100 mm off the central axis (toward upstream; see Figure 2a for an example) to ease manufacturing. Nonetheless, it was still in the range of direct impinging, since the nozzle diameter (D) was 150 mm. Furthermore, because the exhaust fan would be extracting mixed gas and air, the ultimate impinging position on the specimen would be slightly off-center and toward downstream. However, compared to the impinging velocity, the in-chamber airflow was much lower, at approximately 1.9 m/s, which was determined by the flow rate of the exhaust fan. As a result, when the nozzle was adjusted toward the center, the angle iron was wrapped with layers of silica fabric cloth, and the thermal performance of this makeshift insulation method could be evaluated. The second position was 200 mm downstream in relation to the longitudinal center of the specimen. In this case, because the angle iron was not directly impinged, the effects of the core material and other thermal bridges on the insulation performance of the panels could be examined. Meanwhile, the bridge effects of the angle iron, covered or uncovered, were also designated for testing. It should also be pointed out that an area larger than the nozzle diameter (D = 150 mm) on the interior surface would be at a high temperature because the jet was at high velocity.

### 2.3. Measurement Arrangements

**Figure 4:**
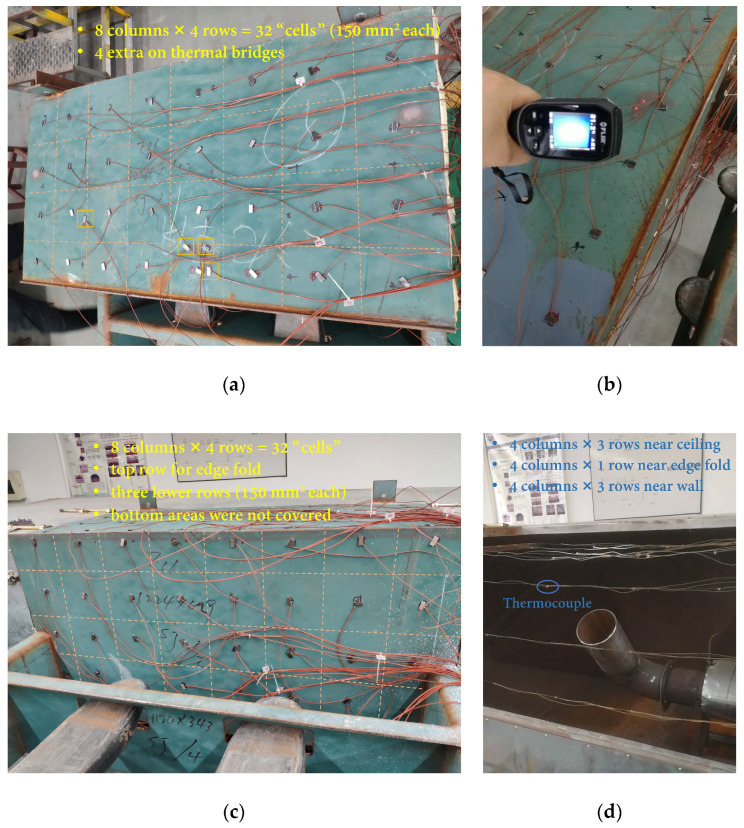
Thermocouple arrangements for interior and exterior surfaces of ceiling and wall panels: (**a**) T-type thermocouples on exterior surface of ceiling panel (specimen 8); (**b**) checking for thermal bridges using a FLIR camera during a pre-experiment; (**c**) T-type thermocouples on exterior surface of wall panel (specimen 8); and (**d**) K-type thermocouples near the two interior surfaces.

The temperature distributions along with the two interior (heating) and two exterior (opposite-side) surfaces of a testing specimen were measured using 97 precalibrated thermocouples wired to three data acquisition systems (Fluke 2638A Hydra Series III, Fluke Corporation, USA), as shown in Figure 4, at 3 s intervals. All thermocouples were kept in the same spot, except for the ones on the thermal bridges.

As illustrated in Figure 4, a hypothetical evenly spaced grid was previously determined to measure the temperature on both exterior surfaces. On the exterior surface of the ceiling panel (see Figure 4a), 32 thermocouples were used, each one placed at the center of a 150 mm^2^ cell. Before formally testing a newly placed specimen for the first time, a pre-experiment is conducted, during which the invisible thermal bridges at normal temperature become more visible. A FLIR camera (see Figure 4b) was also used to confirm and pin down the locations. After the pre-experiment, up to four channels can be set to measure the temperature of the thermal bridges on the exterior surface depending on the specimen (see Figure 4a).

On the exterior surface of the wall panel, as demonstrated in Figure 4c, a top row of eight thermocouples was moved upwards, intended to measure the temperature near the edge fold, while the rest of the thermocouples were placed in 150 mm^2^ cells. The bottom part of the wall panel, which was away from the heating source, was not covered.

Since the two inner surfaces would be impinged by high-temperature gas, K-type thermocouples were used, and the magnet method was no longer possible. As shown in Figure 4d, instead, an L-shaped frame was installed inside the chamber and all 28 thermocouples were prepositioned before the specimen was fitted with the chamber opening. Three rows, i.e., 12 thermocouples in total, were hung below the ceiling at the same height; the fourth row (4 thermocouples) was near the edge fold, and another three rows were behind the wall panel. It should be emphasized that the thickness varied among the eight specimens. Although the frame can be slightly adjusted, it was impractical to guarantee that the thermocouples were all closely adjacent to the intended surface. Therefore, the thermocouples inside the chamber were used to measure near-surface air/gas temperature, while the ones outside were used to measure surface temperature.

Room temperature during each test was recorded using a T-type thermocouple as well to represent the ambient temperature of the SIPs. All data were analyzed using MATLAB^®^ R2019b. When part of the data that came from different acquisition systems was not synchronized in terms of the timeline, it was interpolated, considering the measurement intervals were small (around 3 s) in 30 min, which was deemed to be a baseline with a safety margin, since as a common practice the panels should be able to sustain a heating source for 15 min at most.

### 2.4. Standard Procedure and Testing Design

In this study, SIPs were tested under an impinging jet at a high temperature, which can be considered an extreme testing condition. Since no testing standards were available, the following procedure, which was adopted from an early study [45] for the experiments:

- The locations of the 28 and 64 thermocouples on the interior and exterior surfaces, respectively, were double-checked before the specimen was fitted with the chamber using a forklift.
- The three data acquisition systems for thermocouples, as well as the pitot device, were turned on and started recording.
- The exhaust fan was turned on and the frequency was tuned to 40 Hz, at which the in-chamber airflow was approximately at 1.9 m/s.
- A pre-experiment was scheduled. Before turning on the burner, the air blower for the gas burner was turned on and tuned to 8 Hz. Then, the temperature of the gas that came out of the burner, or the supplying temperature, was controlled and increased to approximately 200–400 °C, which was measured using a thermocouple near the nozzle exit.
- After heating the specimen for a few minutes, the burner was turned down. Afterward, the FLIR camera was used to locate thermal bridges. The specimen was left for self-cooling. Later, additional thermocouples were in close contact with the thermal bridges.
- When the temperature of the specimen approached room temperature, the burner was turned on again. The supplying temperature was adjusted to the designated level, approximately 550 °C, usually within minutes by virtue of a preheated burner. The reading on the pitot device was converted to a gas flow rate based on an estimated density. This time, the time for starting the burner, would be regarded as time zero for the following test.
- The burner was run for 30 min before being turned down. The exhaust fan was kept running to accelerate the cooling process. When it was safe to proceed, the specimen was removed from the chamber. Temperature measurements were examined. The physical deformation of the specimen was checked and documented.
- If another round of tests was needed, the specimen was fitted with the chamber again. In some cases, the specimen was repaired or modified, e.g., by covering the angle iron using silica fabric cloth, before the next experiment.
- The testing order was not based on the specimen ID number. As part of the effort to test the capability of the burner in terms of supplying high-temperature gas, and to develop this standard procedure, specimen 6 was tested first and eventually tested more than 10 times, during which the temperature of the supplying gas was initially at 450 °C and gradually increased to 550 °C. The rest of the specimens were tested at least twice under an impinging jet of around 550 °C.

The overall testing design and thermal bridges under examination using thermocouples are summarized in Table 2.

### 2.5. Heat Flux Analysis

The specimens combined affordable materials and convenient fabrication methods, while possible thermal bridges were put to the test. The nature of the heat transfer was three-dimensional, considering the impingement area was small compared to the panel surface area. Nonetheless, as given in Equation (1), the heat flux of each specimen was analyzed in a simplified manner, assuming one-dimensional heat conduction was taking place and reached a steady state, and both surfaces were assumed to be under convection. It should be noted that the inner surfaces of the specimen were in contact with a tilted impinging jet.

(1) $$q=\frac{T_{f1}-T_{f2}}{\frac{1}{h_{1}}+\sum_{i=1}^{n}\frac{L_{i}}{\lambda_{i}}+\frac{1}{h_{2}}}$$

Here, q is the heat flux of the panel (W·m^−2^), and$\;T_{f1}$ and $T_{f2}$ are the temperature of the hot (impinging jet) and cold (room air) fluids, respectively (°C). In this work, $T_{f1}$ was taken as the average temperature of the mixture of fume and air measured at the interior surface of the ceiling or wall panel; $h_{1}$ and $h_{2}$ are the impinging and convective heat transfer coefficients on each side (W·m^−2^·°C^−1^); $L_{i}$ is the thickness of each layer of the panel (m); $\lambda_{i}$ is the thermal conductivity of each material (W·m^−1^·°C^–1^) and was linear-fitted using available data on 25 °C and 500 °C given by the suppliers, summarized in Table 3. Since $\lambda_{i}$ is temperature-dependent, it is estimated based on the thickness of each material and linear temperature distribution is assumed within the panel.

In addition, Equation (2) was recommended to estimate $h_{1}$ for the impinging jet from a round nozzle [46,47]:

(2) $$\frac{\bar{Nu}}{Pr^{0.42}}=\frac{2D\left(1-2.2D/2r\right)}{r\left[1+0.2\left(\frac{H}{D}-6\right)\left(D/2r\right)\right]}\left[Re^{1/2}{\left(1+0.005Re^{0.55}\right)}^{1/2}\right]$$

where $\bar{Nu}$, Pr, and Re are Nusselt, Prandtl, and Reynold numbers of the impinging jet, respectively ($\bar{Nu}=\frac{h_{1}D}{\lambda }$, $Pr=\frac{v}{\alpha }$, $Re=\frac{VD}{v}$), all dimensionless. The whole panel should be taken when determining $\bar{Nu}$, therefore, lumped variables should be used, i.e., $\lambda =\frac{L}{\sum^{\;}\left(L_{i}/\lambda_{i}\right)}$ and $L=\sum^{\;}L_{i}$; r is the position on the impingement surface (m); α is the thermal diffusivity of the panel (m^2^·s^−1^); H is the distance between the jet and the inner surface of a panel (m); and v is the kinematic viscosity, m^2^·s^−1^.

Equations (3) and (4) were recommended to determine $h_{2}$ based on two scenarios: (1) a lower hot surface (i.e., the exterior surface of the ceiling panel) and (2) a vertical surface (i.e., the exterior surface of the wall panel), respectively [47,48,49].

(3) $$\bar{Nu}=0.52Ra^{1/5}$$

(4) $$\bar{Nu}={\left\{0.825+\frac{0.387Ra^{1/6}}{{\left[{\left(1+0.492/Pr\right)}^{9/16}\right]}^{8/27}}\right\}}^{2}$$

Here, Ra is the dimensionless Rayleigh number, $Ra=\frac{g\alpha_{v}\left[T\left(x=L\right)-T_{f2}\right]l^{3}}{\alpha v}$; $\alpha_{v}$ is the volumetric thermal expansion coefficient (K^−1^); l is the characteristic length: the ratio of surface area to perimeter was used for ceiling panel and height was used for the wall panel (m); and T(x=L) is the estimated average temperature on the exterior surface (°C), which was determined by Equation (5).

(5) $$T\left(x=L\right)=T_{f2}+q\frac{1}{h_{2}}$$

Based on Equations (2)–(5), iterations were taken to compute $h_{1}$, $h_{2}$, and T(x=L). The orders of magnitude for $h_{1}$ and $h_{2}$ were determined at approximately 10^4^ and 10^1^ W·m^−2^·s^−1^, respectively, among all tests.

Another two issues should be pointed out. First, as discussed in Section 3.2, a steady state was not fully reached after 30 min in some cases. As a result, Equation (1) may overestimate the temperature (as in a steady state) on the exterior surface after exposing the impinging jet for 30 min. Second, as summarized in Table 1, many fasteners were potential thermal bridges, which may accelerate local or even overall heat conduction. The implications of the effects of fasteners on heat flux are addressed in Section 3.4. The angle iron representing the hanging bracket can act as a fin made of steel; however, to simplify the analysis, it was ignored in the heat transfer model, but its effect is discussed.

### 2.6. Uncertainty Analysis

The maximum errors of a single K-type or T-type thermocouple are ±0.5 °C ($u_{T_{K}}$) and ± 1.1 °C ($u_{T_{T}}$), respectively. For a given single scan by the data acquisition system, 24 and 32 measurements using K-type and T-type thermocouples are needed to obtain the temperature profiles on the interior and exterior surfaces, respectively. The uncertainty of temperature can be determined by Equations (6) and (7), assuming the errors are all average distributed. $u_{K}$ and $u_{T}$ were calculated at 1.41 °C and 3.59 °C:

(6) $$u_{K}=\sqrt{\overset{24}{\underset{j=1}{\sum }}{\left(\frac{u_{T_{K}}}{\sqrt{3}}\right)}^{2}}$$

(7) $$u_{T}=\sqrt{\overset{32}{\underset{j=1}{\sum }}{\left(\frac{u_{T_{T}}}{\sqrt{3}}\right)}^{2}}$$

where $u_{K}$ and $u_{T}$ are the uncertainty of measurements of temperature profiles of the interior and exterior surfaces of the panels, respectively (°C).

The uncertainties were not determined for heat flux calculations as some of the variables, such as $h_{1}$ and $h_{2}$, were estimated using empirical equations in the literature. To address this issue, the heat flux was calculated for each testing, and no averaging data were used or presented.

### 2.7. Major Limitations

This study was part of the effort to help the industry develop panels used in ships. The panel designs and experimental setup were largely constrained to meet the practical conditions. As a result, major limitations from a research point of view were required by the industry, while it would be better to study the heat conduction of the panels in a simplified way. The limitations included, first, the angle iron and the inclination of the jet nozzle. Second, the testing duration was limited to 30 min to include a practical margin when some of the panels were not in a steady state (additional discussions are provided in Section 3). Third, radiation was not calculated in this study due to a lack of information, such as the internal temperature of the chamber, which could be large. To make the tests comparable, all tests were conducted consecutively with similar room conditions and testing configurations. Additionally, while the heat flux value of each test was estimated using surface temperature, which was considered to be a result of all forms of heat transfer, additional thermal properties, such as overall heat transfer coefficient, etc., were not determined. Fourth, although damages were part of outcomes of the testing, the changes of the thermal performance due to damages after impingement were not quantified, which can be useful in practice, as demonstrated by a similar study [50]. Lastly, the structural performance of the panels was not measured in this study.

## 3. Results and Discussion

### 3.1. Effects of Nozzle Position and Angle Iron

**Figure 5:**
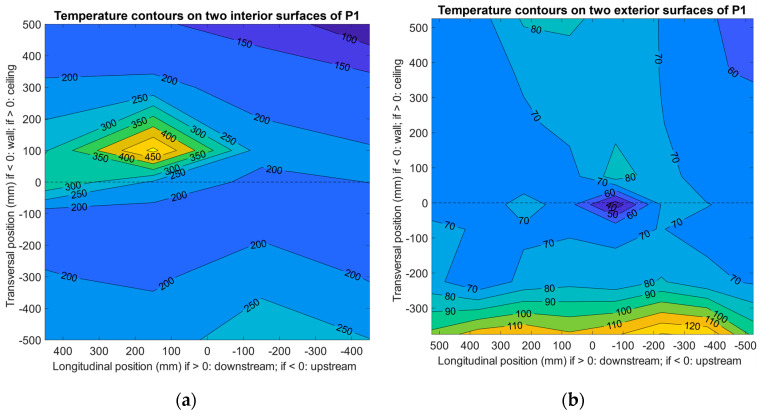

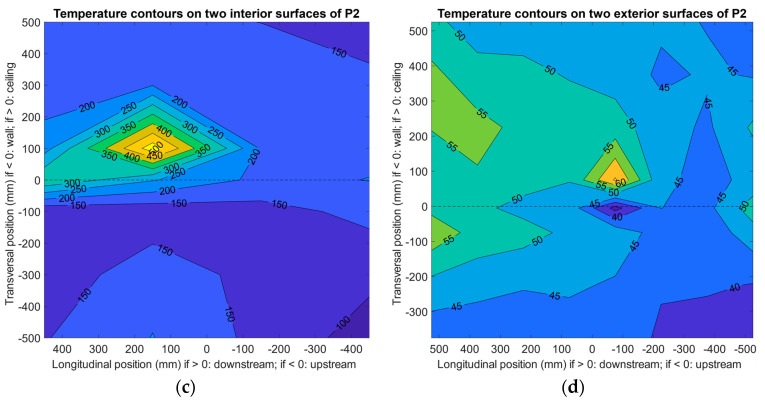
Effects of bare and covered angle irons on the thermal performance of Specimen 1 using a nozzle downstream after impinging for 30 min (data were interpolated by grid measurements): (**a**) case P1, interior surfaces; (**b**) case P1, exterior surfaces; (**c**) case P2, interior surfaces; and (**d**) case P2, exterior surfaces.

Since cases P1 and P2 both used Specimen 1 and the major difference was whether the angle iron was bare (P1) or covered (P2), they were selected to demonstrate the effects of insulating the angle iron on the thermal performance of the whole specimen after being impinged for 30 min, and the results are summarized in Figure 5. In Figure 5, the data were interpolated by grid measurements, while the additional measurements on thermal bridges on the exterior surfaces were excluded because the interpolating algorithm needed to be performed on a matrix. However, since both cases used the same specimen, the fabrication method being tested, in terms of bolts and nails, was the same (therefore, it was not compared or discussed in this section).

It can be found that the area of the high-temperature zone on the interior surface of the ceiling panel (see Figure 5a,c) conformed with the position of the jet nozzle, which was 200 mm downstream in relation to the specimen’s central axis. In this circumstance, the angle iron was not directly impinged, or the jet heavily impinged and heated the specimen. The maximum temperature on the ceiling interior exceeded 510 °C for the two cases, while the opposite locations on the exterior surface reached approximately 55–70 °C, due to the 40 mm thick insulation core (polycrystalline filaments). The temperature was significantly lower on the surfaces of the wall panel compared to the ceiling panel. Case P2 was heated at a slightly higher temperature: the maximum temperature measured near the ceiling interior was around 525 °C. However, the location close to the angle iron on the exterior surface indicated only a mild temperature elevation (see Figure 5d) after being exposed to the high-temperature jet compared to the temperature on the rest of the exterior surface. The major contributor was the insulation cloth on the angle iron. To physically secure the cloth, multiple layers were used, and air was captured in the gaps between layers. It may not be easy to directly quantify the thermal properties of the cloth, even though the effect was certain, by comparing to case P1, during which a clear thermal bridge could be observed, as the maximum temperature on the exterior surface near the angle iron exceeded 90 °C (more information in Section 3.3). The unusual high-temperature area near the lower edge of the wall panel (see Figure 5b) was due to the gap caused by an unmatched specimen–chamber structure, from which a small quantity of high-temperature fume escaped. A provisional solution using additional insulation material to fill the gap was adopted for later testing.

**Figure 6:**
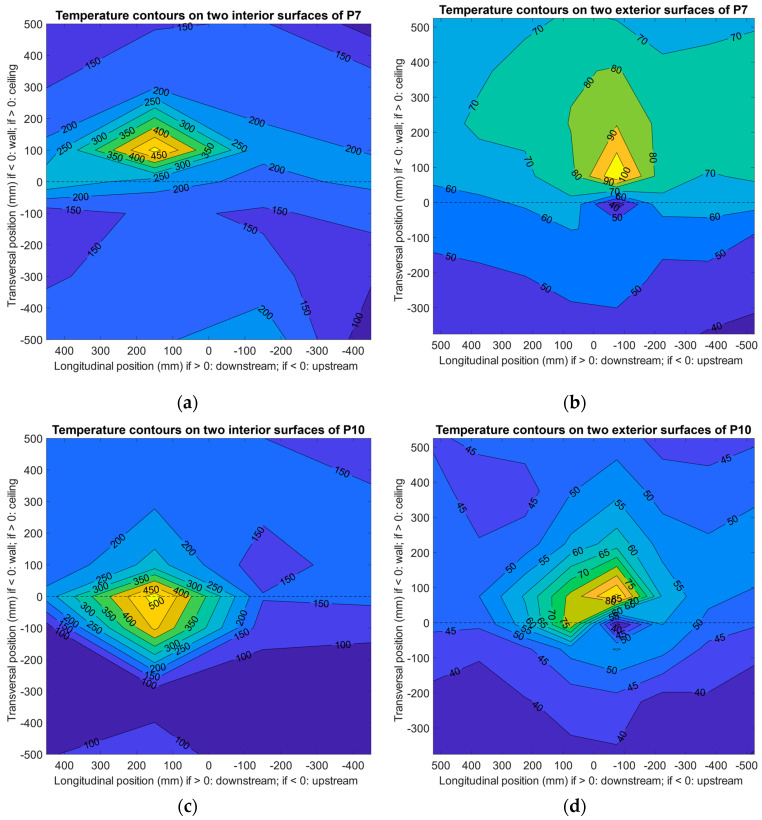
Effects of bare and covered angle irons on the thermal performance of Specimen 3 using a nozzle downstream and center, respectively, after impinging for 30 min (data were interpolated by grid measurements): (**a**) case P7, interior surfaces; (**b**) case P7, exterior surfaces; (**c**) case P10, interior surfaces; and (**d**) case P10, exterior surfaces.

Cases P7 and P10 were also selected to illustrate the effect of nozzle position on the thermal performance of Specimen 3 after being impinged for 30 min, and the results are summarized in Figure 6. For P10, the longitudinal nozzle position, i.e., the impinging position, was aimed at the center of the specimen with the cloth-covered angle iron, and for P7 it was 200 mm from the center toward downstream with the angle iron bare to the impinging jet. Again, data on the additional measurements of thermal bridges on the exterior surfaces were excluded from Figure 6 to generate temperature contours; they are included and discussed in Section 3.3.

Similar to the pattern of cases P1 and P2 (see Figure 5), when the nozzle was downstream (P7, see Figure 6a), the temperature was significantly higher on the ceiling panel than the wall panel; this was expected based on the experimental design, which was to test the insulation of the normal area of the specimen with a “unusual” thermal bridge (e.g., a bare piece of angle iron as an accessory to the panel) nearby. However, in this case, the nearby thermal bridge was distinct; the maximum temperature on the ceiling exterior surface reached almost 110 °C (see Figure 6b and Section 3.3). Since the volume of the angle iron was not small, neither was the bridging effect. A larger area of the exterior surface of the ceiling panel was under high temperature, although the wall panel seemed normal and the insulation core was as effective as it was in cases P1 and P2 (the same insulation material was used as the core of P1–P10).

On the other hand, among the nine cases, P10 used a central-positioned nozzle to test the specimen with a cloth-covered angle iron under direct impinging. Interestingly, in this case the angle iron acted as a block and prevented the jet from immediately heating the panel. Nonetheless, the maximum temperature measured on the inner space was still as high as 500 °C, indicating that the jet still hit the panel near the edge fold toward downstream (see Figure 6c) at high speed (otherwise a larger high-temperature area may be expected) after passing the angle iron. On the exterior surfaces, the maximum temperature (near 100 °C) was recorded on the location opposite the angle iron stretching toward the inner surface, whereas the rest of the panel was not severely heated (see Figure 6d). Thus, in this case, direct heating at the thermal-protected angle iron did not elevate the overall surface temperature of the panel on the other side.

### 3.2. Effects of Fastener Methods

During the testing of each case the specific focus was on the effects of fastener methods (bolts, nails, battens, seams, etc.), and it was suggested that various effects could be found among specimens.

**Figure 7:**
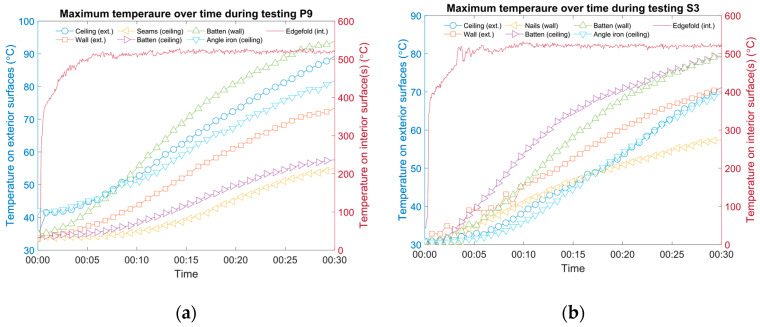
Examples of cases where surface temperatures opposed to fasteners were higher compared to the angle iron and monitoring grid: (**a**) case P9, specimen 3 and (**b**) case S3, specimen 5.

Figure 7 shows two examples (cases P9 and S3) where the surface temperature opposite the fasteners was higher than that of the angle iron and the monitoring grid on the exterior surface. In both cases, battens were used to fix the insulation core: polycrystalline filaments for P9 (Specimen 3) and silica aerogel for S3 (Specimen 5). The height of the battens was different: two half-thickness battens were used for P9 (one batten was as thick as one layer of core material, and the other was connected using long rivets; see Figure 1 for illustration) and two full-thickness battens for S3 (one batten was as thick as two layers of core material, and only short rivets were needed). For both cases, the angle iron was capped using cloth, yet the temperature on the exterior surface still approached 80 °C (see Figure 7a) and 70 °C (see Figure 7b) after being directly impinged for 30 min (with the nozzle in the center in both cases). It should be noted that the initial temperatures on the two panel surfaces were slightly different (approximately 3 °C). However, the recorded temperatures focusing on the battens were higher compared to the angle iron, indicating a hidden thermal bridge, which can be worse than a cloth-covered angle iron, in both cases. In addition, a straight seam between two pieces of steel on the interior surface of the ceiling panel of P9 was deliberately designed to be tested, as well as a spot for nails on S3; both tested negative for registering as additional thermal bridges during the testing period. Last but not least, even though the maximum temperature on the inner surface occurred at a steady state after around 10 min, the rate of temperature increase on the outer surface was still noticeable after 30 min. However, testing for a longer period was not planned or carried out since the scenario was considered unlikely in practice.

**Figure 8:**
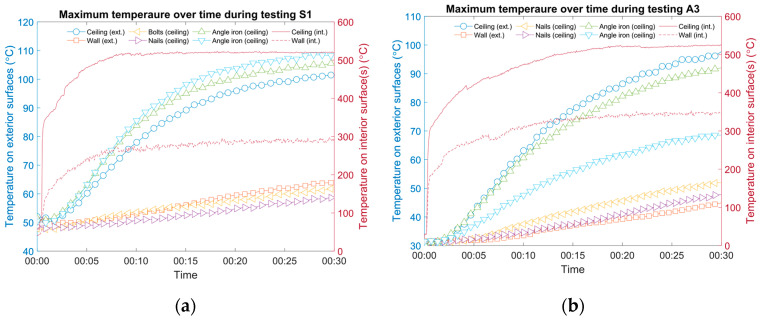
Examples of cases where surface temperature opposite fasteners varied: (**a**) case S1, specimen 4 and (**b**) case A3, specimen 7.

Although the temperature discrepancies between battens, nails, and seams were large for cases P9 and S3, according to Figure 7, they can be even larger, as the examples in Figure 8 show.

For case S1, the temperature of the measurement spots of nails and bolts on the ceiling increased only about 10 °C after testing for 30 min (see Figure 8a). On the contrary, the temperature substantially increased by more than 50 °C to surpass 100 °C on the exterior surface of the angle iron. Although it should be repeated that while the angle iron was bald, the thermal bridge effect it caused seemed limited, as possible nearby fasteners were not hugely affected. The insulation core of aerogel was effective. For case A3, similarly, when the iron was not shielded by layers of cloth, the temperature of different fasteners could vary broadly (see Figure 8b). In this case, the temperature on the outer surface of the ceiling panel was elevated by approximately 70 °C, while it increased by 15 °C for the ceiling nails and 10 °C for the outer surface of the wall panel. Another practical reason was that not all fasteners were thoroughly heated by the jet, while some were deliberately fabricated to be close to the impingement area.

### 3.3. Cross-specimen Comparisons

**Figure 9:**
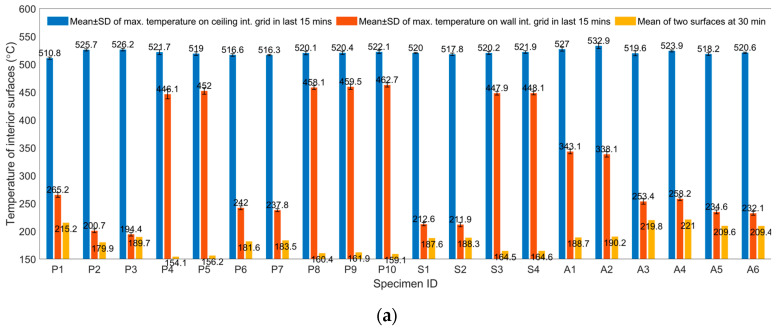

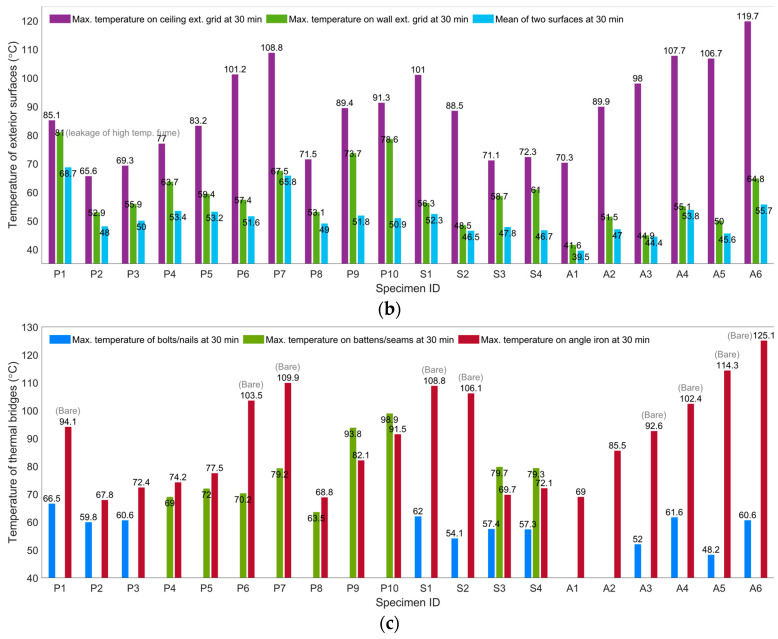
Cross-specimen comparisons: (**a**) grid temperature on two interior surfaces; (**b**) grid temperature on two exterior surfaces; and (**c**) thermal bridge effects of various fabrication methods.

In addition to the discussions on specific cases in previous sections, cross-specimen comparisons were conducted among all 20 cases from the three aspects of grid temperature on the two interior surfaces, grid temperature on the two exterior surfaces, and thermal bridge effects of various fabrication methods, as summarized in Figure 9. When computing average temperature on exterior surfaces, data from thermocouples that were close to the edges of the panel were omitted to minimize the effect of leaked fumes from the chamber. Leakage can be ignored, except in case P1.

The following conclusions can be made, as illustrated in Figure 9a. Based on the design and operation of the chamber, it was difficult to guarantee that each case would be tested under identical conditions, considering temperature fluctuations in the room (ranging from 28 to 33 °C, which can be considered small compared to the temperature differences between the interior and exterior surfaces of the SIPs caused by the impinging jet), the burning efficiency of the burner, whether the chamber and specimen were perfectly fitted (if not, it was hard to modify in the field), the thickness of the panel, etc. Nevertheless, the measured maximum temperature on the interior surface of the ceiling panels was relatively consistent (521.1 ± 4.6 °C), despite the nozzle being aimed at the center in 9 out of 20 cases (see Table 2) and direct impinging on the panel was thus blocked. Furthermore, during the last 15 min of each testing, the impinging temperature was also fairly steady (standard deviation was small). On the other hand, the interior surface temperature of the wall panel evidently correlated to the nozzle position, i.e., when the angle iron was not blocking the impinging jet, the ceiling directly impinged while the wall panel suffered less severe heating (maximum temperature was around 250 °C or less). When the iron half-intercepted the jet, the direction of the jet changed toward the wall panel, causing the temperature on the interior to increase to 330 °C for A1 and A2, and even up to 462 °C for P10. Moreover, the average temperature of the two interior surfaces, which signaled the total heat transfer of the panel, was computed for each case. A recognizable pattern can be found showing that leaving the iron bare elevated the overall inner temperature beyond 200 °C; otherwise, the inner surface temperature could be lower than 150 °C on average.

The comparisons of the exterior surfaces and thermal bridges are shown in Figure 9b,c, respectively. As discussed previously (Figure 8), after being exposed to high temperature for 30 min, many specimens were not in a steady state in terms of heat transfer on the exterior surface, and the following conclusions can be drawn. First, a few layers of cloth on the angle iron would be helpful to lower the temperature on the exterior surface only for the short term. Adhesive substances used between the iron and cloth would lose efficacy under high temperature for a long time, resulting in less effective insulation performance (see cases P8–P10 and A1–A2. S1–S2 were exceptions). Second, by comparing first-round tests with either a central-aligned nozzle (cases P4, S3, and A1) or a downstream-positioned nozzle (cases P2, P6, S1, A3, and A5), the thermal performance of aluminum silicate was better than that of polycrystalline filaments and silica aerogel, in terms of the average temperature on the outside surface. The highest temperature on the ceiling may be affected by the angle iron, therefore, it was not treated as a major indicator. P1 was excluded due to leakage of high-temperature fumes. Third, while a bare angle iron was an indisputable thermal bridge, a covered one can still be one as well. A few layers of cloth may work adequately for a short period. Among the tested specimens, bolts and nails showed a less explicit thermal bridge effect. Either full-thickness or half-thickness battens can be a concern. The total surface area of battens was larger than that of nails or bolts. Fourth, as long as the adhesive was working (normally under approximately 400–500 °C) and the gap was hard to penetrate, even under a high-speed impinging jet, seams would be fine. Finally, a panel based on Specimens 1 (e.g., case P2) and 5 (e.g., S3) can be a priority, considering the thermal performance, material availability, weight, convenience for mass production, etc.

### 3.4. Heat Flux and Implications

**Figure 10:**
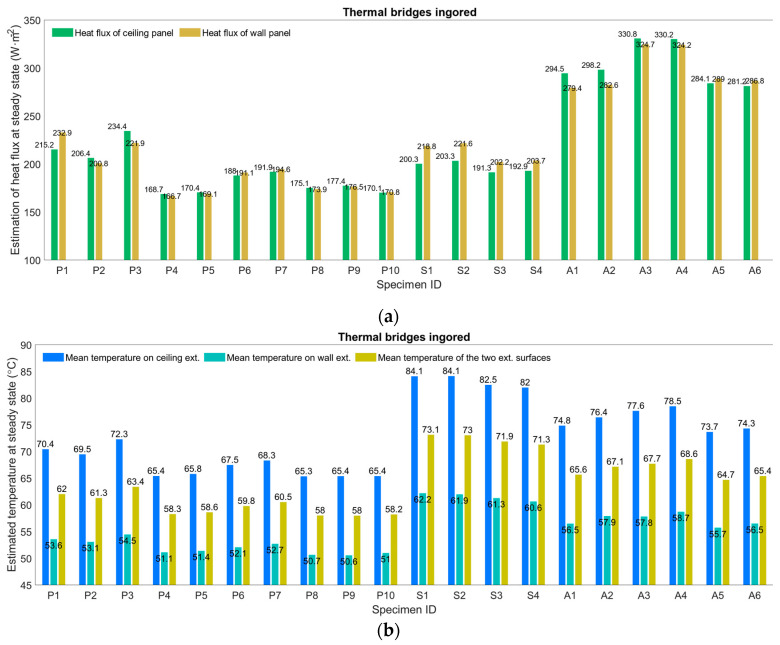
Estimations of (**a**) heat flux and (**b**) exterior surface temperature at a steady state.

Based on Equations (1)–(5), heat flux and exterior surface temperature for each case at a steady state were estimated and are shown in Figure 10. Thermal bridges were ignored during the estimations.

It can be concluded from Figure 10a that in most cases heat flux was higher for the ceiling panel than the wall panel. Exceptions can also be found, partly because the model used to calculate heat flux was semiempirical, so deviations were possible. In addition, overall, panels using aluminum silicate as the insulation core resulted in high heat flux, while the other two types of insulation produced low heat flux. This seems contradictory to the findings based on temperature on the outside surface in Figure 9b. This can partly be explained as follows: First, it took various amounts of time for the heat conduction of the specimens to reach a steady state, and the surface temperature at the two exterior surfaces at steady state depended on additional variables, such as room temperature and convective heat transfer. According to Figure 10b, the estimated average surface temperature was around 58–73 °C. In all cases, the ceiling panel was hotter than the wall panel, since the majority of fumes were targeted at the ceiling interior surface. At steady state, panels with an aerogel core seemed least effective at thermal insulation. According to Table 3, the thermal conductivity of aerogel was the lowest at 25 °C and 500 °C, among the three tested materials. We should keep in mind that only 20 mm thick aerogel was used, while 40 mm was used for the other two materials. By comparing Figure 9 and Figure 10, we can observe that most of the estimated surface temperature was higher than the measured temperature of the grid, which is normal, considering that in many cases a steady state was not reached. A quick calculation based on Fourier number, Fo ($Fo=\alpha t/L^{2}$), shows that hours would be needed for the heat conduction to reach a quasi-steady state. Second, in testing, the surface temperature was affected by thermal bridges, which were ignored when estimating the heat flux. Based on Figure 9, much of the measured temperature on the thermal bridges was already higher than the estimations of surface temperature at a steady state, suggesting that the fabrication methods and materials play a vital role in the use of panels, as well as in their design. Heating the thermal bridges for a relatively short period, the elevated temperature on the other side of the panel would surpass that of a bridge-free panel under a steady state, for which much more time may be needed.

### 3.5. Physical Damage

While physical durability (including damage) was not the focus of this work, general observations were concluded from post-experiment inspection for future reference. First, as reported earlier, the temperature at the impingement area was around 520 °C and higher than the normal working condition of adhesives, and as a result, the adhesive substances lost efficacy in many cases and became brittle. Second, when using silica fabric cloth as an overall cover for the panel, the insulation performance was generally intact under short-term impinging, although the surface of the impingement area was covered with a powder-like white substance. Third, when using the cloth to cover a large thermal bridge, the insulation performance can be compromised after being exposed to high-temperature fumes for 30 min or longer. This may be mitigated by changing the adhesive to another practical fabrication method.

## 4. Conclusions

In this work, a panel chamber matching setup was designed to investigate the thermal performance of sandwich panels under an impinging jet at 550 °C for 30 min. Three types of low-cost materials (polycrystalline filaments, silica aerogel, and aluminum silicate) that are commonly used in the shipbuilding industry were selected as the insulation core. Various fabrication methods (bolts, nails, battens, and seams) as well as a section of angle iron were also included as potential thermal bridges. A total of eight specimens were manufactured and tested in 20 cases. The temperature on the four surfaces of each panel was measured in a structured grid fashion, and additional measurements were conducted to record temperature changes on the thermal bridges. Heat flux was also estimated for all cases to estimate the elevated temperature on the opposite surface of the panel under free convection.

Major conclusions include the following: First, based on cross-specimen comparisons, the maximum temperature of the impinging fumes measured on the interior (or heating) surface of the panel was consistent among all cases, while the maximum grid temperature on the exterior surface under convection varied, ranging from 41 to 120 °C, depending on the insulation core and, more importantly, thermal bridges. Second, among the thermal bridges, the angle iron was the most significant. Most of the highest temperatures measured on the exterior surfaces were caused by a section of bare angle iron. In addition to the large volume and surface area of the angle iron, the thermal conductivity of the steel can be two to three orders of magnitude higher than that of the polycrystalline filaments or silica aerogel, causing a rising of overall heat transfer of the SIPs significantly. As a provisional improvement, covering the iron with a few layers of cloth could decrease the elevated temperature by up to 50 °C. However, post-experiment inspection confirmed the drawbacks of adhesive, therefore the improvement may be temporary. Bolts and nails were less effective as thermal bridges, while in-core battens were more effective. Third, the increased temperature on the thermal bridges after 30 min surpassed the estimated surface temperature at a steady state, which could be hours long. The heat flux was estimated for all cases and ranged from 167 to 331 W·m^−2^. Last, from a holistic perspective, two specimens exhibited promising overall performance, although further improvements to the panel design are still needed, especially in the method to minimize the thermal bridge effect of large accessories or components of the panel.

## Figures and Tables

**Table 1:** Materials and fabrication methods used in specimens.

ID	Structure Layer	Core	Heating Layer	Panel	Fastener Methods
1	Steel, grade EH36, 7 mm thick	Two layers of polycrystalline filament, together 40 mm thick	Stainless steel, grade 316L, 1 mm thick	Ceiling	Two rows of bolts (ϕ10 mm) + one row of welding nails (ϕ5 mm) + adhesive used on the seam
				Wall	Two rows of welding nails (ϕ5 mm)
2				Ceiling	Two full-thickness battens: one near the end and one near and under the seam; short rivets connecting the batten and the heating layer of steel
				Wall	Two full-thickness battens: one at the bottom with rivets, one near the edge fold
3				Ceiling	Two half-thickness battens: one near the end and one near and under the seam; long rivets connecting the batten and the heating layer of steel
				Wall	Two half-thickness battens: one at the bottom with rivets, one near the edge fold
4		Two layers of silica aerogel, together 20 mm thick		Ceiling	Two rows of welding nails (ϕ5 mm) + one row of bolts (ϕ10 mm)
				Wall	Two rows of welding nails (ϕ5 mm)
5				Ceiling	Two full-thickness battens: one near the end and one near and under the seam; short rivets connecting the batten and the heating layer of steel
				Wall	Two rows of welding nails (ϕ5 mm) + two full-thickness battens: one at the bottom with rivets, one near the edge fold
6		Two layers of aluminum silicate, together 40 mm thick	Silica fabric cloth, approx. 1 mm thick	Ceiling	Two rows of welding nails (ϕ5 mm) + one stainless-steel compacting bar + adhesive to fix the overlap of two layers of cloth
				Wall	Two rows of welding nails (ϕ5 mm) + one compacting bar
7				Ceiling	One compacting bar + three rows of welding nails (ϕ5 mm); the additional row of nails (compared to specimen 6) fixed the overlap of two layers of cloth
				Wall	Two rows of welding nails (ϕ5 mm) + one compacting bar
8				Ceiling	Two rows of welding nails (ϕ5 mm) + two compacting bars; additional bar with nails (compared to specimen 6) covered the overlap of two layers of cloth
				Wall	Two rows of welding nails (ϕ5 mm) + one compacting bar

**Table 2:** Overall testing design and thermal bridges of interest.

Case ID. ^1^	Specimen ID.	Nozzle Longitudinal Position	Number of Repetitions	Thermal Bridges Under Examination				
				Bolts	Nails	Seam	Battens (With Rivets)	Angle Iron
P1	1	Downstream	1	Ceiling	Ceiling			Bare
P2, P3			2	Ceiling	Ceiling			Covered
P4, P5	2	Center	2				Ceiling + wall, full-thickness	Covered
P6, P7	3	Downstream	2			Ceiling		Bare
P8–P10		Center	3				Ceiling + wall, half-thickness	Covered
S1, S2	4	Downstream	2	Ceiling	Ceiling			Bare
S3, S4	5	Center	2		Wall		Ceiling + wall, full-thickness	Covered
A1, A2	6	Center	2					Covered
A3, A4	7	Downstream	2		Ceiling			Bare
A5, A6	8	Downstream	2		Ceiling			Bare

^1^ Letters indicate main core materials: P, polycrystalline filaments; S, silica aerogel; A, aluminum silicate.

**Table 3:** Physical properties of materials used in panel specimens.

Material	λ (W·m^−1^·°C^−1^)
EH36 steel	−0.0244*T* + 45.369
Polycrystalline filaments	0.00020*T* + 0.027
Silica aerogel	0.000086*T* + 0.019
Aluminum silicate	0.000242*T* + 0.032
